# Lysosomal Drug Sequestration Mediated by ABC Transporters and Drug Resistance

**DOI:** 10.3390/pharmaceutics17101255

**Published:** 2025-09-24

**Authors:** Petr Mlejnek

**Affiliations:** Department of Anatomy, Faculty of Medicine and Dentistry, Palacky University Olomouc, Hnevotinska 3, 77515 Olomouc, Czech Republic; mlejnek_petr@volny.cz

**Keywords:** mechanisms of drug resistance, lysosomal mediated drug resistance, lysosomal ABC transporters

## Abstract

**Background**: Drug resistance (DR) mediated by ABC transporters in the cytoplasmic membrane has been one of the best studied mechanisms of DR in vitro. More recently, it has also been suggested that ABC transporters expressed on lysosomal membranes could increase the sequestration of anticancer drugs in lysosomes, thereby reducing their concentration at target sites, and causing DR. Unfortunately, convincing evidence that such a DR mechanism actually exists is lacking, even in the case of in vitro experiments. **Methods**: This hypothetical study using simplified models evaluates the effect of ABC transporter-mediated accumulation of anticancer drugs in lysosomes on their concentration at target sites under standard in vitro conditions. **Results**: Calculations show that an ABC transporter resident on the plasma membrane must create and maintain a relatively small concentration gradient between extracellular space and the target site to reduce the drug concentration at the target site by, for example, half. In contrast, if a lysosomal ABC transporter is to also halve the concentration of the drug at the target site, then it must create and maintain a huge concentration gradient between lysosomes and target sites. It is very likely that massive accumulation of drugs in lysosomes would have a negative effect on the function of the lysosomes themselves. **Conclusions**: The results of this hypothetical study strongly suggest that the mechanism of DR mediated by lysosomal ABC transporters is questionable, as it requires enormous accumulation of the drug in lysosomes, which would likely also impair their function. Therefore, it is highly unlikely that this hypothetical DR mechanism could actually be utilized by tumor cells to defend against the cytotoxic effects of chemotherapy in vitro.

## 1. Introduction

Drug resistance (DR) is a major factor in cancer recurrence despite significant successes with targeted chemotherapy [1,2]. Among the best-studied mechanisms of DR in vitro is reduced intracellular drug concentration mediated by ABC transporters residing in the plasma membrane. Indeed, in laboratory experiments, it is easy to demonstrate a causal relationship between the expression of the relevant ABC transporter, reduced drug concentration and, consequently, its reduced cytotoxic effect [3,4]. Unfortunately, these promising results have not been translated into clinical practice [5].

In the last three decades, it has been suggested that ABC transporters could also mediate DR as influx pumps if they reside in the membranes of cytoplasmic vesicles, very often identified as lysosomes. It is assumed that ABC transporters functioning on lysosomal membranes could increase the sequestration of antitumor drugs in lysosomes, thereby reducing their concentration at target sites and thus causing DR. The first studies to suggest that intracellular ABC transporters may contribute to overall DR involved ABCB1 (P-glycoprotein) and anthracyclines [6,7]. Later, Yamagishi et al. suggested that ABCB1 is located not only on the cytoplasmic membrane but also in lysosomes, where it may enhance lysosomal sequestration of drugs and thus also contribute to the development of DR [8]. This mechanism was not observed for all drugs that are ABCB1 substrates, but only for those that exhibit hydrophobic weak base properties [8]. Rajagopal and Simon showed that ABCC1 (MRP1) activity on lysosomes is sufficient to confer drug resistance to doxorubicin, whereas alkalization of lysosomal pH is not [9]. These studies seem convincing, but they rest on assumptions whose validity needs to be critically verified. First, the quantitative distribution of drugs (i.e., anthracyclines, doxorubicin or daunorubicin) is evaluated according to its fluorescence intensity in each compartment. It is assumed that the fluorescence intensity of anthracyclines is directly proportional to their concentrations in each compartment. Based on this assumption, the high fluorescence intensity of lysosomes and the low fluorescence intensity of the nucleus are interpreted as “the ability of lysosomes” to reduce the concentration of the drug in the nucleus, i.e., at the target site. However, it is important to realize that this interpretation is based on assumptions that are completely contrary to reality and therefore lead to incorrect conclusions [10]. Indeed, more precise quantitative determination of the content of anthracyclines in cells or their compartments (e.g., in the nucleus) requires the use of more sophisticated methods [11,12]. Second, although both ABCB1 and ABCC1 can be localized in lysosomes, their functionality there is questionable, as lysosomes are the site of their degradation [13,14].

In contrast, the functional expression of ABCA2, ABCA3, ABCA5, ABCB6, ABCB9, and ABCD4 on lysosomes is not questionable [14]. Several studies have implicated ABCA2 transporter in drug resistance phenotype of cancer cells. Importantly, these studies mostly relay on correlation between extent of *ABCA2* expression and cell sensitivity to particular anticancer drug in vitro [15,16,17]. Similarly, there is indirect evidence that ABCA5 may mediate DR in cancer cells [18]. In mammalian cells, ABCA6 expression has been shown to be associated with increased resistance to cadmium [19]. This effect has been explained by the ability of ABCB6 to accumulate cadmium in lysosomes. However, the details of the resistance mechanism are far from clear [19]. Conflicting results exist for the ABCB9 transporter. For example, increased sensitivity to paclitaxel was achieved by reducing ABCB9 expression by microRNA-24 in resistant MCF-7 cells [20]. In contrast, its increased expression in malignant NCI-H2452 cells resulted in increased sensitivity to cisplatin [21]. ABCA3 is somewhat better studied in relation to DR. Thus, Chapuy et al. demonstrated that intracellular ABCA3 transporter confers drug resistance toward a broad spectrum of anticancer substances in HEK293 and HL-60 cells by lysosomal drug sequestration in vitro [22]. Later, the same research group showed that the intracellular ABCA3 transporter may contribute to imatinib resistance by facilitating its lysosomal sequestration in chronic myeloid leukemia cells [23]. However, there is no direct evidence that DR is caused by reduced drug concentration at the target site due to increased lysosomal sequestration mediated by ABCA3 [22,23]. Recently, Panneerselvam et al. described cisplatin resistance in non-small cell lung cancer (NSCLC) mediated by a mechanism dependent on ABCD4 expression [24]. The details of the mechanism are not yet known [24]. In clinical practice, intracellular ABC transporters are not considered mediators of DR. However, the expression of some of them (ABCA3, ABCA5 and ABCB9) is used as a prognostic factor [14]. Examples of drugs and heavy metals that are sequestered in lysosomes are listed in Table 1.

A careful analysis of the published results shows that it is unclear to what extent (if at all) intracellular ABC transporters may actually contribute to the development of DR in vitro. The DR mechanism mediated by lysosomal ABC transporters, unlike DR mechanisms mediated by other factors, can also be studied computationally. This hypothetical study analyzes the quantitative aspects of lysosomal drug sequestration mediated by ABC transporters and its possible contribution to the development of DR in vitro.

## 2. Material and Methods

A detailed description of the models and equations used for the calculation is given below. The graphs were made using the SigmaPlot 11.0 software package (Systat Software Inc., San Jose, CA, USA). Parameters for model to calculate the drug concentration gradient mediated by ABC transporters:

### 2.1. ABC Transporter in the Lysosomal Membrane Forms Drug Concentration Gradient Between Lysosomes and Target Sites

This thought experiment is performed in a closed system containing a suspension of tumor cells in growth medium. The total system volume (V_T_), consisting of the volume of growth medium (i.e., the volume of extracellular space; V_ECS_) and the total cell volume (V_CT_), is 10 mL:

V_T_ = V_ECS_ + V_CT_ = 10 mL (1)

Total cell volume (V_CT_) includes cell volume without lysosomes (volume of the target sites; V_TS_) and lysosomal volume (V_L_):

V_CT_ = V_TS_ + V_L_ (2)

Combination (1) and (2) gives:

V_T_ = V_ECS_ + V_TS_ + V_L_ = 10 mL (3)

A drug is added to the system that is a substrate for the ABC transporter. We further assume that:

(a) The volume of the drug is negligible and does not affect the total volume of the system (V_T_);
(b) Drug is uncharged molecule that can pass freely through the cell membranes;
(c) Only two interactions of the drug are considered: (i) the passive diffusion of uncharged molecules <=> and (ii) active transport mediated by ABC transporter (Figure 2a,b);
(d) Unless otherwise stated, the number of cells is constant (total number of cells: 5 × 10^6^; cell density: 0.5 × 10^6^/mL);
(e) Cells have an ideal spherical shape with an average diameter of 18 μm;
(f) Cells contain one compartment—lysosomes, which volume represents 5% of the total cell volume (unless otherwise stated);
(g) The volume of cells and lysosomes does not change with drug accumulation.

The molar amount of drug in the system can be described as follows:

n_T_ = n_ECS_ + n_TS_ + n_L_ (4)

Indices ECS, TS and L stands for the extracellular space (growth medium), target sites (intracellular space without lysosomes) and lysosomes, respectively.

The molar amount of drug in “compartment i”:

n_i_ = V_i_ C_i_ (5)

Combination of (4) and (5) gives:

n_T_ = V_ECS_C_ECS_ + V_TS_C_TS_ + V_L_C_L_ (6)

If the ABC transporter is inactive, then the drug concentration in all compartments is the same due to free diffusion (Figure 2a) and the following applies:

C_ECS_ = C_TS_ = C_L_ (7)

If the ABC transporter in the lysosomal membrane is active, the drug accumulates in lysosomes and therefore drug concentration in lysosomes is higher than that at target sites. Due to the free diffusion of the drug between the extracellular space and the target sites, its concentration between these compartments is equalized. Thus, the decrease in the concentration of the drug at the target sites is inextricably linked to its simultaneous and equal decrease in the extracellular space. For this reason, the relationship between concentrations in individual compartments can generally be described as follows:

C_L_ > C_TS_ = C_ECS_ (8)

The decrease in drug concentration at target sites, as a result of ABC transporter activity, can be expressed using a factor x, which takes on the following values: x ≤ 1, x > 0. Note: If x = 1, then there is no loss of drug at the target site. The change in drug concentration at the target sites due to ABC transporter activity can then be expressed as follows:

C_TS_ = xC^0^_TS_ (9)

where C^0^_TS_ is the initial equilibrium concentration of the drug at the target sites established by free diffusion without ABC transporter expression (Note: since we consider the drug as an uncharged molecule, the pH gradient between lysosomes and target sites does not contribute to its accumulation in lysosomes)

Combining Equations (8) and (9) we obtain Equation (10):

xC^0^_TS_ = xC^0^_ECS_ (10)

where C^0^_ECS_ is the initial equilibrium concentration of the drug in extracellular space (growth medium) established by free diffusion without ABC transporter expression

The reduced molar amount of drug at the target site and the extracellular space (growth medium) due to the activity of ABC transporters can be expressed as follows:

n_ECS_ + n_TS_ = V_ECS_xC^0^_ECS_ + V_TS_xC^0^_TS_ (11)

Combining (10) and (11), we can modify the relationship as follows:

n_ECS_ + n_TS_ = (V_ECS_ +V_TS_)xC^0^_TS_ (12)

In lysosomes, on the contrary, there is an increase in the molar amount of the drug, which can be expressed as follows:

n_L_ = V_L_C^0^_L_ + (V_ECS_ +V_TS_)(1 − x)C^0^_TS_ (13)

where C^0^_L_ is the initial equilibrium concentration of the drug in lysosomes established by free diffusion without ABC transporter expression

The increased concentration of the drug in lysosomes due to the activity of ABC transporters can then be expressed as follows:

C_L_ = n_L_/V_L_ = [V_L_C^0^_L_ + (V_ECS_ + V_TS_)(1 − x)C^0^_TS_]/V_L_ (14)

The change in drug concentration between two compartments can be expressed either as a difference (C_L_ − C_TS_) or as a ratio (C_L_/C_TS_), which is more suitable for large changes, as in our case. Using Equations (9) and (14), we get (15):

C_L_/C_TS_= {[V_L_C^0^_L_ + (V_ECS_ +V_TS_)(1 − x)C^0^_TS_]/ V_L_}/ xC^0^_TS_ (15)

### 2.2. ABC Transporter in the Cell Membrane Forms Drug Concentration Gradient Between Extracellular Space and Target Sites

Similarly here, this thought experiment is performed in a closed system containing a suspension of tumor cells in growth medium. The total system volume (V_T_), consisting of the volume of growth medium (i.e., the volume of extracellular space; V_ECS_) and the total cell volume (V_CT_), is 10 mL:

V_T_ = V_ECS_ + V_TS_ = 10 mL (16)

A drug is added to the system that is a substrate for the ABC transporter. We further assume that:

(a) The volume of the drug is negligible and does not affect the total volume of the system (V_T_);
(b) Drug is uncharged molecule that can pass freely through the cell membranes;
(c) Only two interactions of the drug are considered: (i) the passive diffusion of uncharged molecules <=> and (ii) active transport mediated by ABC transporter (Figure 2a,c);
(d) Unless otherwise stated, the number of cells is constant (total number of cells: 5 × 10^6^; cell density: 0.5 × 10^6^/mL);
(e) Cells have an ideal spherical shape with an average diameter of 18 μm;
(f) Cells contain no compartments (therefore, the total volume of cells = volume of the target sites).

The molar amount of drug in the system can be described as follows:

n_T_ = n_ECS_ + n_TS_ (17)

Indices ECS and TS stand for extracellular space and target sites, respectively.

The molar amount of drug in “compartment i”:

n_i_ = V_i_ C_i_ (5)

Combination of (17) and (5) gives:

n_T_ = V_ECS_C_ECS_ + V_TS_C_TS_ (18)

If the ABC transporter is inactive, then the drug concentration in all compartments is the same due to free diffusion (Figure 2a) and the following applies:

C_ECS_ = C_TS_ (19)

If the ABC transporter in the cell membrane is active, the drug is transported from the target sites to the extracellular space (growth medium), and therefore drug concentration in the extracellular space is higher than that at target sites. The relationship between drug concentrations in the aforementioned compartments can generally be described as follows:

C_ECS_ > C_TS_ (20)

The decrease in drug concentration at target sites, as a result of ABC transporter activity, can be expressed using a factor x, which takes on the following values: x ≤ 1, x > 0. Note: If x = 1, then there is no loss of drug at the target site. The change in drug concentration at the target sites due to ABC transporter activity can then be expressed as follows:

C_TS_ = xC^0^_TS_ (9)

where C^0^_TS_ is the initial equilibrium concentration of the drug at the target sites established by free diffusion without ABC transporter expression

The reduced molar amount of drug at the target site due to the activity of ABC transporters can be expressed as follows:

n_TS_ = V_TS_xC^0^_TS_ (21)

In extracellular space, on the contrary, there is an increase in the molar amount of the drug, which can be expressed as follows:

n_ECS_ = V_ECS_C^0^_ECS_ + V_TS_(1 − x)C^0^_TS_ (22)

where C^0^_ECS_ is the initial equilibrium concentration of the drug in extracellular space (growth medium) established by free diffusion without ABC transporter expression

The increased concentration of the drug in extracellular space can then be expressed as follows:

C_ECS_ = n_ECS_/V_ECS_ = [V_ECS_C^0^_ECS_ + V_TS_(1 − x)C^0^_TS_]/V_ECS_ (23)

In this case, we will also express the change in drug concentration between the extracellular space and target sites as the ratio (C_ECS_/C_TS_) to easily compare both variants of ABC transporter localization (see above). Using Equations (9) and (23), we get (24):

C_L_/C_TS_ = {[V_ECS_C^0^_ECS_ + V_TS_(1 − x)C^0^_TS_]/V_ECS_}/xC^0^_TS_ (24)

## 3. Results and Discussion

**Figure 1:**
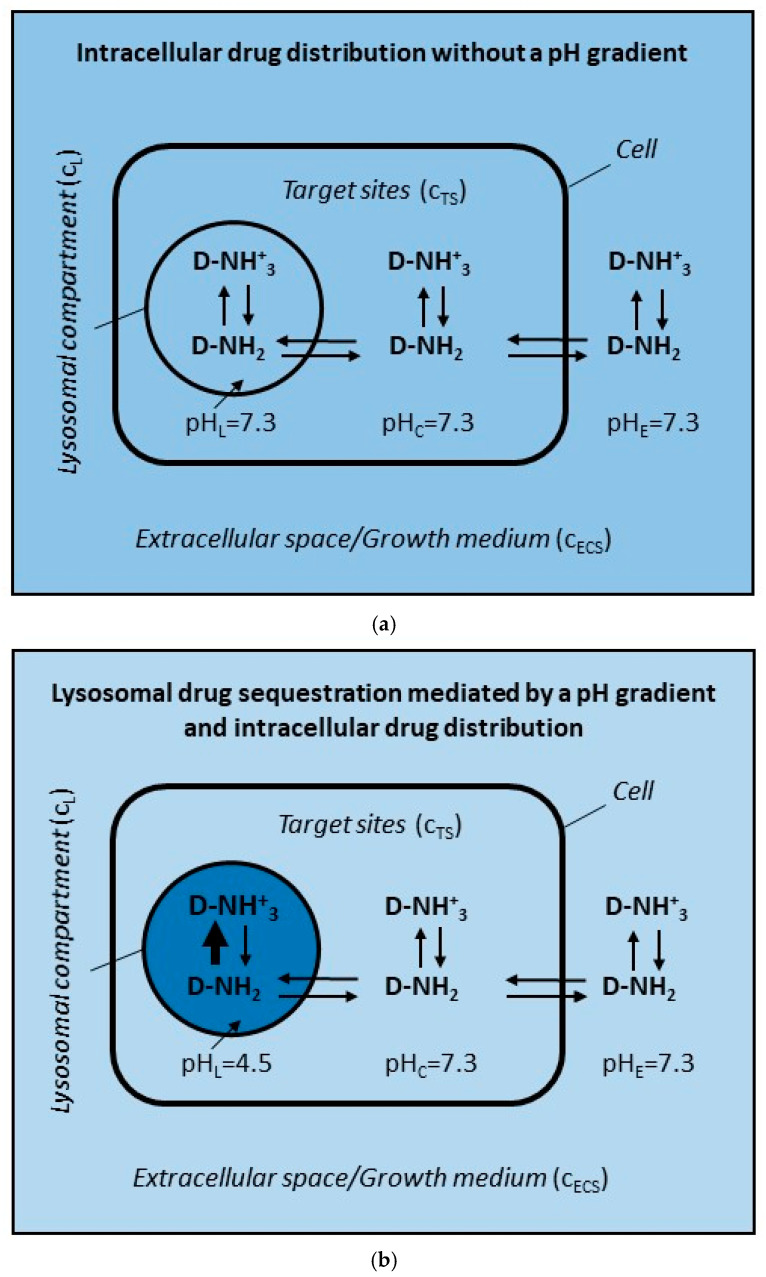
A model for analyzing the effect of the pH gradient on the concentration of hydrophobic weak-base drug at target site in cancer cells. It is assumed that: (i) the uncharged form of the drug can freely pass through cell membranes; (ii) only two interactions of the drug are considered, the Henderson–Hasselbach equilibrium ↑↓ and the passive diffusion of uncharged molecules <=>. C_L_, C_TS_, and C_ECS_ are abbreviations for drug concentrations in lysosomes, at the target sites, and in the extracellular space, respectively. (**a**) Without a pH gradient between lysosomes and cytosol. C_L_ = C_TS_ = C_ECS_. (**b**) With a pH gradient between lysosomes and cytosol. C_L_ >> C_ECS_ = C_TS_. The darker the color, the higher the drug concentration and vice versa.

**Figure 2:**
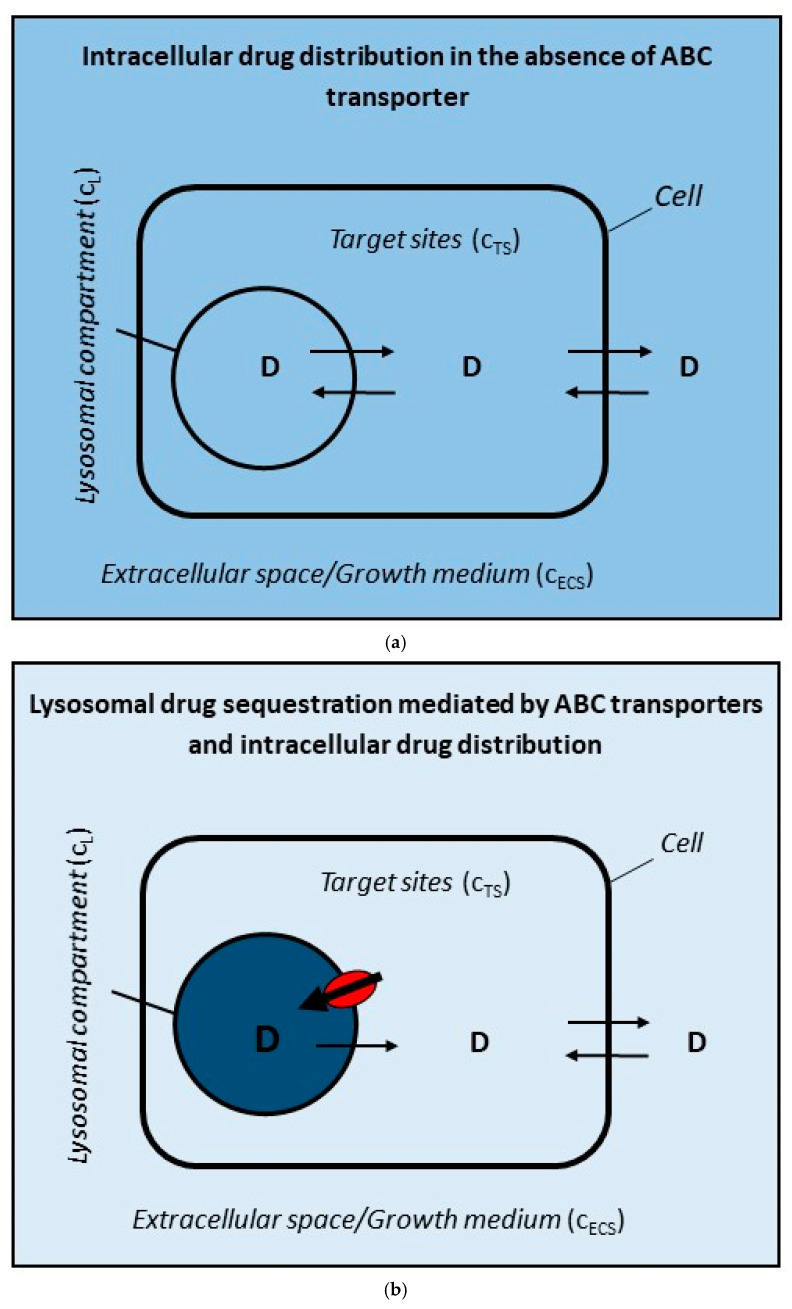

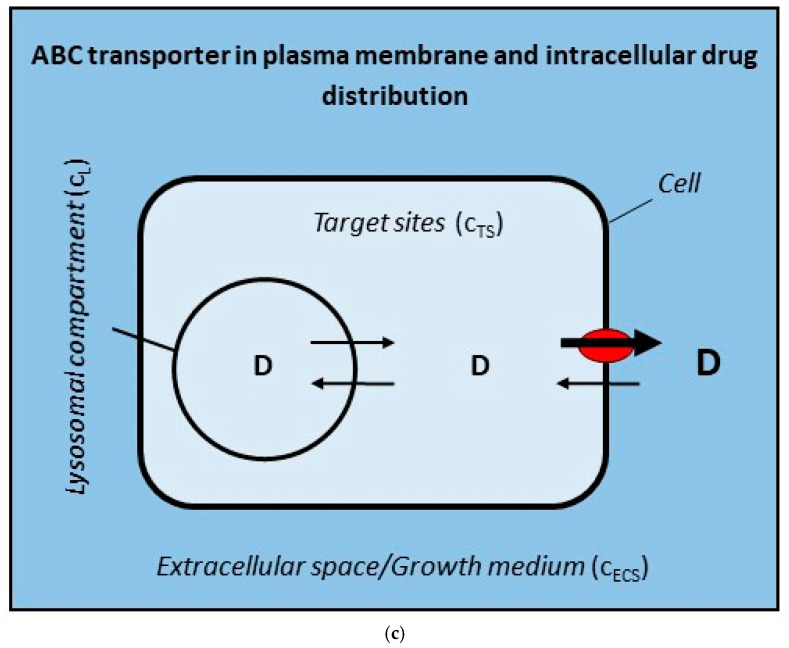
A model for analyzing the effect of an ABC transporter functioning in different cellular compartments on the concentration of anticancer drugs at the target site. It is assumed that: (i) drug is a hydrophobic molecule without a charge; (ii) it can freely pass through cell membranes (passive diffusion) <=>; (iii) drug serves as a substrate for ABC transporter. C_L_, C_TS_, and C_ECS_ are abbreviations for drug concentrations in lysosomes, at the target sites, and in the extracellular space, respectively. (**a**) Drug distribution in the cancer cell without an ABC transporter. C_L_ = C_TS_ = C_ECS_. (**b**) Drug distribution in a cancer cell with an ABC transporter residing in lysosomal membrane. Drug transport is oriented towards the inside of lysosomes. C_L_ >> C_ECS_ = C_TS_. (**c**) Drug distribution in a cancer cell with an ABC transporter in plasma membrane. Drug transport is oriented out of the cells. C_L_ = C_TS_ < C_ECS_. The darker the color, the higher the drug concentration and vice versa. ABC transporter is marked in red.

In addition to a wide range of physiological and pathological functions, lysosomes are often associated with the possible emergence of DR through alteration in the intracellular distribution of some anticancer drugs [26,27,28,29]. The basic mechanism is increased accumulation (i.e., sequestration) of the drug in lysosomes, either due to the pH gradient between lysosomes and cytosol (Figure 1), or due to the activity of lysosomal transporters, most often ABC transporters (Figure 2b), which leads to a decrease in its concentration at target sites and thus to a decrease in its cytotoxicity and the development of DR [6,28]. The DR mechanism mediated by ABC transporters in the cytoplasmic membrane is also associated with reduced drug concentration at target sites (Figure 2c). Unlike the DR mechanisms described above, this mechanism is very well understood, at least under in vitro conditions [3,4]. The common feature of these DR mechanisms, namely the reduced drug concentration at target sites, allows us to make some useful comparisons—see below.

Lysosomal sequestration of weak-base drugs driven by the pH gradient between the cytosol and lysosomes is considered as one of the putative DR mechanisms (Figure 1b) [28]. However, the causal link among lysosomal drug sequestration, its reduced concentration at target sites, and the development of DR is hard to find in published studies. In contrast, there are studies including those from our laboratory that clearly show that lysosomal sequestration of weak-base anticancer drugs has no or only marginal effect on their concentration at the target site [10,30,31,32,33]. My recent theoretical analysis of this phenomenon, consistent with these experimental results, clearly shows that under standard conditions in in vitro experiments, lysosomal sequestration of weak-base drugs leads to a decrease in their concentration of less than 5% [34]. The main reason for this is the incommensurable volume ratios between the site of drug sequestration, i.e., lysosomes, and the other components of the cell system, i.e., cytosol (target sites) and extracellular space (growth medium). The details are given elsewhere [34].

Lysosomal sequestration of uncharged hydrophobic drugs or hydrophobic weak-base drugs driven by ABC transporters residing in the lysosomal membrane represent another controversial mechanism of DR (Figure 2b). Similarly, it is difficult to find a causal link among lysosomal drug sequestration, its reduced concentration at target sites, and the development of DR in published studies [14,35]. DR due to the activity of ABC transporters in lysosomal membranes, can be estimated computationally in in vitro experiments (Figure 2b). For this purpose, several simplifying assumptions have been adopted (see Section 2). The most important of these are related to the physico-chemical properties of anticancer drugs themselves. We consider only those that are substrates of ABC transporters and are either hydrophobic molecules without charge or hydrophobic weak-base molecules a percentage of which, depending on the pH, is always uncharged [28,36]. In general agreement with other researchers, we further assume that the uncharged form of drugs can freely pass through cell membranes. The consequence is that the concentration of the uncharged form of the drug is the same in the cytosol (target sites) and in the extracellular space (growth medium). As a result, the decrease in the concentration of the drug in the target sites is inextricably linked to the decrease in its concentration in the extracellular space (Figure 2b). Thus, the same rules apply here as in the case of lysosomal sequestration driven by the pH gradient between lysosomes and the cytosol (see above, Figure 1).

**Figure 3:**
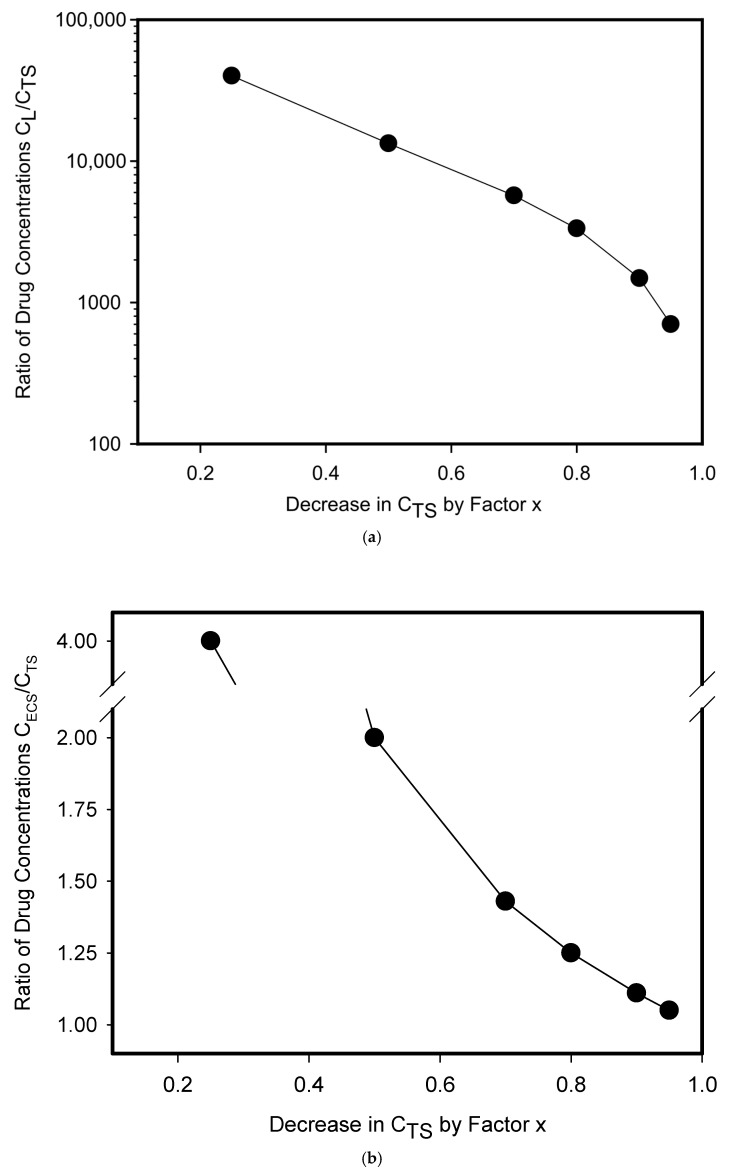
Expression of ABC transporter in different cellular compartments and formation of drug concentration gradient. The basic system parameters are as follows: total volume of the system V_T_ = 10 mL; cell density 0.5 × 10^6^/mL; cell diameter 18 μm; lysosomal volume represent 5% of cell volume. C_L_, C_TS_, and C_ECS_ are abbreviations for drug concentrations in lysosomes, at the target sites, and in the extracellular space, respectively. (**a**) ABC transporter in lysosomal membrane. The graph shows the relationship between the decrease in drug concentration at the target site (C_TS_) by factor x (C_TS_ = xC^0^_TS_; where C^0^_TS_ is the initial equilibrium concentration of the drug at the target sites established by free diffusion without ABC transporter expression) and the resulting concentration gradient between lysosomes and the target site (C_L_/C_TS_). X takes on the following values: x ≤ 1, x > 0. Note: If x = 1, then there is no decrease in drug concentration at the target site. (**b**) ABC transporter in plasma membrane. The graph shows the relationship between the decrease in drug concentration at the target site (C_TS_) by factor x (see above) and the resulting concentration gradient between the extracellular space and the target site (C_ECS_/C_TS_). Note: If x = 1, then there is no decrease in drug concentration at the target site.

Calculations based on the above assumptions under standard in vitro experimental conditions (see Section 2) for drugs that belong to the category of uncharged hydrophobic substances and that are simultaneously substrates of ABC transporters show that even a small decrease in drug concentration at the target site (C_TS_) requires an enormous increase in drug concentration in lysosomes (C_L_; Figure 2b and Figure 3a). This means that the ABC transporter residing in the lysosomal membrane must create and maintain a large concentration gradient between lysosomes and target sites (Figure 2b and Figure 3a). In general, the change in drug concentration between two compartments can be expressed either as a difference (C_L_ − C_TS_) or as a ratio (C_L_/C_TS_), which is more suitable for large changes such as in our case (Figure 3a). The decrease in drug concentration at target sites, as a result of ABC transporter activity, can be expressed using a factor x: C_TS_ = xC^0^_TS_; where C^0^_TS_ is the initial equilibrium concentration of the drug at the target sites established by free diffusion without ABC transporter expression (See Section 2; Figure 3a). This situation is incomparable to the functioning of the ABC transporter residing in the cytoplasmic membrane (Figure 2c and Figure 3b). Here too, the change in drug concentration between the extracellular space and target sites is expressed as the ratio (C_ECS_/C_TS_) and the decrease in drug concentration at the target site is expressed by the factor x (see Section 2). The same concentration change at the target site (C_TS_) requires the creation and maintenance of a relatively small concentration gradient between extracellular space and target sites (Figure 2c and Figure 3b). For example, if the concentration of the drug at the target site is reduced by half (C_TS_ = 0.5C^0^_TS_), the concentration gradient between lysosomes and target sites increases more than thirteen thousand-fold (C_L_/C_TS_ > 13,000) for a lysosomal ABC transporter (Figure 3a). In contrast, if the concentration of the drug at the target site is reduced by half (C_TS_ = 0.5C^0^_TS_), the concentration gradient between extracellular space and target sites increases approximately twofold (C_ECS_/C_TS_ ≈ 2) for an ABC transporter in the plasma membrane (Figure 3b).

If the drug under study were a substance that belongs to the category of hydrophobic weak bases, then we would reach very similar results. Let us consider the same example as above, i.e., reducing the concentration of the drug at the target site by half (C_TS_ = 0.5C^0^_TS_). Given that pH-gradient-driven lysosomal sequestration of hydrophobic weak-base drugs is capable of reducing its concentration at the target site by a maximum of 5% [34], then it is sufficient for lysosomal ABC transporters to contribute to the overall reduction by 45%, thereby achieving an overall reduction in the drug concentration at the target site to 50%, i.e., by half. A 45% reduction in drug concentration at the target site by lysosomal ABC transporters (C_TS_ = 0.55C^0^_TS_) corresponds to a concentration ratio C_L_/C_TS_ > 10,000 see Section 2).

The above examples very well illustrate the low efficiency and high energy demand of reducing the drug concentration at the target site through its sequestration by lysosomal ABC transporters, regardless of whether the drug is an uncharged hydrophobic molecule or a hydrophobic weak base. Moreover, it is likely that the excessive accumulation of a drug in the lysosomes would have a number of negative functional consequences [37,38]. In the case of enormous accumulation of hydrophobic weak-base drugs, pH alkalization occurs in the lysosomal lumen with following impaired autophagy, or permeabilization of the lysosomal membrane with subsequent induction of cell death [38,39,40].

**Figure 4:**
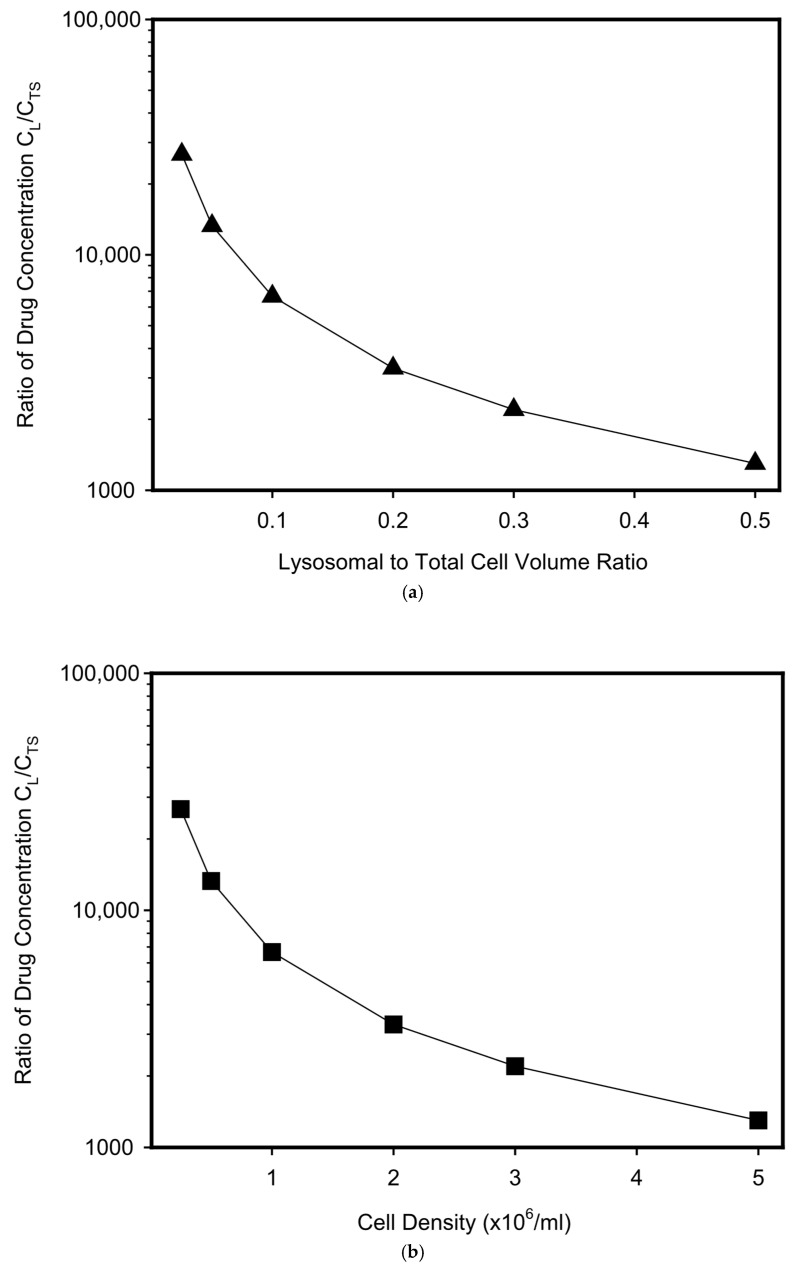
Effect of the volume of lysosomal compartment on the resulting concentration gradient between lysosomes and the target site. The basic system parameters are as above; concentration of the drug at the target site is reduced by half (C_TS_ = 0.5C^0^_TS_). (**a**) Effect of lysosomal volume on the resulting concentration gradient between lysosomes and the target site. Cell density is constant (0.5 × 10^6^/mL); lysosomal volume varies from 2.5 to 50% of cell volume. (**b**) Effect of cell density on the resulting concentration gradient between lysosomes and the target site. Cell density varies from 0.25 to 5.0 × 10^6^/mL; lysosomal volume is constant and represents 5% of cell volume.

The massive sequestration (accumulation) of the drug in lysosomes can be partially reduced by increasing the volume of the lysosomal compartment, either directly by increasing the number or size of lysosomes (Figure 4a), or indirectly by increasing cell density while maintaining the size and number of lysosomes within a single cell (Figure 4b). Although a tenfold increase in the volume of the lysosomal compartment, whether direct or indirect, leads to a tenfold decrease in the magnitude of the concentration gradient between lysosomes and target sites needed to achieve the desired reduction in drug concentration at target sites, its magnitude is still too large to make this DR mechanism effective (Figure 4). Overall, this hypothetical study indicates that lysosomal sequestration of anticancer drugs mediated by lysosomal ABC transporters is a very inefficient way to reducing their cytotoxicity. The calculations offer a possible explanation for why no convincing experimental evidence has been found that lysosomal sequestration of these drugs mediated by lysosomal ABC transporters could lead to DR, even in the case of the ABCA3 transporter, which is probably the best studied [22,23,35].

The hypothetical model used in this work leads to the conclusion that the mechanism of DR mediated by lysosomal ABC transporters is questionable because it requires an unrealistic amount of drug accumulation in lysosomes, which would likely impair their function. Therefore, it is highly unlikely that this putative DR mechanism would actually be utilized by tumor cells in defense against the cytotoxic effects of chemotherapy in vitro.

## Figures and Tables

**Table 1:** Examples of drugs and heavy metals that are sequestered in lysosomes.

Transporter	Weak-Base Drugs	Hydrophobic Drugs	Other Drugs/Heavy Metals
ABCA2	mitoxantrone * [16]	Estradiol *, estramustine [15]	Cisplatin * [17]
ABCA3	daunorubicin *, mitoxantrone *, vincristine *, imatinib [22]	etoposide * [22]	-
ABCA5	dasatinib * [18]	-	thiosemicarbazone derivative * [18]
ABCB1 ^†^	daunorubicin, doxorubicin, vincristine, vinblastine, imatinib, gefitinib [25]	tacrolimus, colchicine, teniposide, podophyllotoxin, paclitaxel [25]	-
ABCB6	-	-	cadmium [19]
ABCB9	-	paclitaxel * [20]	-
ABCC1 ^†^	anthracyclines, vincristine, vinblastine, imatinib, gefitinib [25]	flutamide, cochicine, teniposide, paclitaxel [25]	arsenate, arsenite [25]
ABCD4	-	-	cisplatin * [24]

^†^ putative lysosomal transporters [13,14]. * putative substrates [14,15,16,17,18,19,20,22,24].

## Data Availability

The data presented in this study are available in this article.
